# Psychosocial Experiences Related to Dietary Behavior of Japanese Lactating Women: A Qualitative Study

**DOI:** 10.3390/nu15030789

**Published:** 2023-02-03

**Authors:** Kaori Matsuda, Mie Shiraishi, Natsuki Hori, Hanna Horiguchi, Masayo Matsuzaki

**Affiliations:** Department of Children’s and Women’s Health, Division of Health Sciences, Graduate School of Medicine, Osaka University, Osaka 565-0871, Japan

**Keywords:** breastfeeding, dietary behavior, Japanese women, postpartum period, psychosocial experience, qualitative research

## Abstract

Adequate dietary intake during the lactation period is important for breast milk components, postpartum recovery, and physical and mental health. This study aimed to clarify the psychosocial experiences related to dietary behavior around one month postpartum among Japanese lactating women. Semi-structured interviews were conducted with 18 women between February and June 2022 in Osaka, Japan. The data were analyzed using qualitative descriptive approaches. Four core categories were identified. All participants had a [desire to have healthy meals for themselves or their families] to improve their postpartum health, regain their pre-pregnancy body shape, produce sufficient and good-quality breast milk, and keep their families healthy. Some participants, who had [subjective difficulties in getting information on diet and preparing meals] due to insufficient or complicated information and viewing meal preparation as a burden, used [services and support regarding their postpartum diet] to alleviate these difficulties. They had [postpartum-specific appetite and dietary views], including an increased appetite triggered by breastfeeding and postpartum stress and the effects of the dietary changes during pregnancy. Some of these psychosocial experiences were influenced by Japanese traditional customs in the postpartum period and familiar food preferences in the Japanese. Healthcare professionals should consider these experiences when providing tailored dietary guidance.

## 1. Introduction

Lactating women are more likely to be motivated to have a healthy diet, considering the impact of diet on breast milk and their physical recovery after childbirth [1,2,3]. Despite this motivation, 30% of Japanese primiparas felt that they were too preoccupied with the care of their babies to take care of their own diet [4]. Japanese studies reported that lactating women perceived that their psychosocial experiences, such as a lack of postpartum dietary support and a sense of burden due to poor cooking skills and a lack of time, had an influence on their dietary behaviors [2,5]. In fact, 50–90% of lactating women in Japan did not meet their dietary requirements for important nutrients, such as iron, calcium, and vitamins [6,7]. To improve this nutritional status, a randomized controlled trial of individual educational intervention on diet was conducted with Japanese postpartum women; however, the effect of the intervention was limited to increased iron and total dietary fiber intake [7]. While lactating women desire a healthy diet, the results indicate that it is difficult to achieve this goal through educational interventions, suggesting that various psychosocial perspectives need to be considered when providing dietary guidance.

Previous studies in other countries have further indicated that the dietary behaviors of women during the postpartum or lactation period were affected by psychological experiences, such as attitude toward weight management, psychological stress, and their perceptions of their energy needs when taking care of their babies [8,9,10,11,12]. In addition, social experiences have been reported to influence dietary behaviors, including consuming unhealthy convenience foods because of having no time to prepare meals [9,13,14], the financial costs of buying healthy foods [9,13,15], and the lack of accurate information on healthy postpartum diet [10,16]. These psychosocial experiences identified in other countries have not been confirmed in Japanese lactating women. Since attitudes toward diet are generally influenced by culture [15,17,18] and dietary behaviors during the postpartum period can often be influenced by traditional customs [19], Japanese lactating women may have different psychosocial experiences from women in other countries.

In Japan, many women customarily receive support for meal preparation and childcare from family members at their parents’ home or their own home for the first month after childbirth, while others do the housework and childcare by themselves [20,21]. Thus, the circumstances surrounding dietary behaviors around one month postpartum vary from person to person. In addition, women in this period often experience mastitis and other physical or mental discomforts [22,23]. Thus, women in the first month postpartum are likely to be physically and mentally unstable because they are in the process of recovering from childbirth and adjusting to their new lives. Physical and mental health are influenced by dietary intake [24,25,26,27]. For these reasons, we believe that effective dietary guidance and support are especially necessary for this period.

Understanding the various psychosocial experiences related to dietary behavior can help healthcare providers assess the context of lactating women’s dietary behavior and provide dietary advice tailored to individual situations. Therefore, the aim of this study was to clarify the psychosocial experiences related to the dietary behavior of Japanese lactating women around one month postpartum.

## 2. Materials and Methods

### 2.1. Definition of a Key Term

“Dietary behavior” was defined as including the amount of meals, frequency of meals, meal contents, meal time, and meal preparation.

### 2.2. Study Design, Setting, and Participants

A qualitative study was conducted through in-depth, semi-structured interviews. We recruited Japanese women who participated in a prospective cohort study that was conducted at one maternal clinic in Osaka and examined the women’s lifestyles and health during the perinatal period. We selected this clinic because it handles a large number of low-risk deliveries, and the present study aimed to identify common experiences of obstetrically low-risk Japanese women. Women who were breastfeeding in the first month postpartum were included in the present study. Women with a history of mental illness, non-Japanese women, and women who the clinic staff determined to have difficulty participating in the study had already been excluded from the prospective cohort study. In addition, women with dietary restrictions due to diseases were excluded before recruitment for the present study. The participants were selected through purposeful sampling.

### 2.3. Recruitment

A question asking whether women would agree to receive an invitation to an interview was added to the one-month postpartum questionnaire of the prospective cohort study between December 2021 and April 2022. We sent information about the study and an invitation to participate in an online interview via e-mail to all women who agreed to receive them. Their willingness to participate in the study was confirmed based on their reply to the email. The “date of childbirth”, “parity”, “age”, “education”, “marital status”, “family type”, and “body satisfaction at one month postpartum” of the participants were ascertained in advance from the cohort study to schedule the date of the interview and understand the characteristics of the participants. Regarding body satisfaction, the participants were asked, “Are you satisfied with your body shape?” at one month postpartum and could respond with “satisfied”, “neither satisfied nor dissatisfied”, or “dissatisfied”.

### 2.4. Data Collection

Online interviews via Zoom or LINE were conducted individually by the first author between February and June 2022. The interview duration ranged from 25 to 38 min for an average of 30 min. Almost all interviews were conducted via video calls, except when participants refused. We audio recorded the interviews with the consent of the participants.

The interviews were set at 3 months postpartum to enable the participants to recall and share their experiences around the first month postpartum. The interviews followed an interview guide that included the following questions: (1) How did you want to have your diet around one month postpartum? Why did you think so? (2) What was your actual dietary behavior around one month postpartum? (3) Was there anything that prevented you from having the diet you wanted? What was it? (4) Was there anything that helped you achieve the dietary goals you wanted for yourself? What was it?.

### 2.5. Data Analysis

We used a qualitative descriptive approach. The approach aims to comprehensively summarize events in everyday terms and is suitable for describing phenomena straightforwardly [28]. The interview data were transcribed to create a verbatim transcript. Data on the psychosocial experiences related to dietary behavior were extracted as one unit for each semantic content and paraphrased close to the raw data. The paraphrases were recorded and presented using codes. Codes with commonalities were collected to form subcategories, and categories were based on subcategories with similarities. By collecting categories of commonalities, core categories were generated at a higher level of abstraction. The data were immediately analyzed after each interview, with the next interview beginning after the analysis of the previous interview. We ended the interviews after we confirmed that no new codes had emerged, which meant data saturation had been reached.

### 2.6. Trustworthiness

Trustworthiness was ensured based on the method proposed by Lincoln and Guba [29]. All analyses were conducted by the first and second authors. The credibility and confirmability of this study were confirmed through peer debriefing by several researchers specializing in midwifery and member checking. Member checking was performed by sending the results to the participants via e-mail and requesting checks and comments. In addition, the dependability and the transferability were ensured by describing the process of study and results in detail.

### 2.7. Ethical Considerations

The participants were informed of their voluntary participation, confidentiality, the purpose of the study, and publication of the results using explanatory documents. Prior to the start of each interview, the purpose and content of the study were explained in writing and orally, and verbal consent was obtained for participation. In addition, we explained that the participants could have a break to care for or feed their babies. This study was approved by the Research Ethics Committee of Osaka University (No.20525).

## 3. Results

### 3.1. Participants’ Demographic Characteristics

Eighteen women participated in this study. The ages of the participants ranged from 28 to 41 years (mean: 34 years). Nine participants were primiparas, while nine were multiparas (Table 1). All participants were married and lived in a nuclear family unit. Only Participant L worked from home at one month postpartum.

### 3.2. Perceived Psychosocial Experiences Related to Dietary Behavior of Japanese Lactating Women

Four core categories, nine categories, and twenty-five subcategories were identified as psychosocial experiences related to the dietary behavior of Japanese lactating women (Table 2). In the text, core categories are described with square brackets, categories with single quotation marks, subcategories with double quotation marks, and narratives with *italics*. All participants had a [desire to have healthy meals for themselves or their families]. Some participants had [subjective difficulties in getting information on diet and preparing meals], while they used [services and support regarding their postpartum diet] to alleviate these difficulties. All participants recognized [postpartum-specific appetite and dietary views], which were influenced their dietary behaviors, such as amount of meals, frequency of meals, and meal contents.

#### 3.2.1. Desire to Have Healthy Meals for Themselves or Their Families

There were three reasons for the desire to eat healthier: “desire to have good meals for their postpartum body”, “desire to have good meals for breast milk” to contribute to the health and growth of their babies, and “desire to prepare good meals for their family”.

- “Desire to have good meals for their postpartum body”

The participants had a “desire to prevent mastitis” and tried to eat a diet based on Washoku (Japanese traditional cuisine) or reduce the amount of sweet or high-fat foods to prevent mastitis.

*I tried to avoid fatty foods, such as stew, until one or two months after childbirth because I was afraid of getting mastitis. I’ve heard that eating fatty foods can cause mastitis, so I tried to avoid them. (B)*

They had a “risk perception of poor health related to childbirth or postpartum process,” which was a reason for their desire for a healthy diet to improve their own physical condition in the postpartum period, compensate for a lack of sleep due to caring for their babies, and cope with concerns about recovery after a cesarean section.

*I had a crisis about not sleeping well, and I didn’t want to get sick. I thought I had to get enough nutrition (to keep my health). So, I was trying to eat well. (C)*

*My childbirth was an emergency cesarean section. I was very afraid because I had my stomach cut, and I had pain after the cesarean section. I thought I might die. I wanted to live longer, so I had to eat healthy. (G)*

They also recognized the importance of their own health for childcare and had a “desire to remain healthy to care for their babies.”

*I had to work hard for breastfeeding. So I consciously tried to eat iron-rich foods. (R)*

All participants who had a “desire to regain pre-pregnancy weight and shape” were dissatisfied with their body shape. They tried to adjust the content, amount, or time of their meals to regain their pre-pregnancy body weight and shape.

*I thought that I wouldn’t lose weight if I didn’t care about it consciously. After the birth of my third child, I started weighing myself every day and adjusting the amount of food I ate. (H)*

- “Desire to have good meals for breast milk”

The participants had a “desire for the baby to drink good quality breast milk.” For them, a good diet for breast milk meant eating Washoku, foods high in protein and vitamin K, and avoiding junk food and fatty foods.

*I heard that vitamin K is essential for babies. […] I heard that if I take enough vitamin K, it goes directly to my baby. So, I eat natto. (natto: Japanese fermented soybeans high in vitamin K) (Q)*

Some avoided spicy foods, such as curry, because they believed spicy foods would affect the taste of the breast milk. Caffeine and alcohol were also avoided before breastfeeding because they can negatively affect babies.

*I think that caffeine is bad for my baby. I got the information from the internet. […] I heard that babies can’t fully break down caffeine, which makes it harder for them to fall asleep* *. So, I tried to refrain from taking it before breastfeeding. (P)*

All participants who had a “desire to produce a sufficient amount of breast milk” fed their babies both breast milk and formula at one month postpartum. They tried to eat sufficient amounts of food and to eat foods that would improve milk supply, such as rice, rice cakes, soups, and protein-rich foods.

*I tried to eat as much food as possible to produce a lot of breast milk. (I)*

*I heard somewhere that eating rice cakes produces a lot of breast milk, so I tried eating them. (D)*

- “Desire to prepare good meals for their family”

The participants had a “desire to prepare meals for the good health of their families or to maintain regular mealtimes for their families,” which was mentioned by some multiparas and a primipara. They wanted their husbands and older children to have nutritious and balanced meals. The belief that their families would be happier and healthier with the meals they prepared was also a motivator for cooking. They recognized that this had resulted in their own healthy diet.

*When preparing meals for family members, I try to cook with sufficient amounts of vegetables, carbohydrates, and protein. I try to make sure that the meals are well-balanced. […] I cook for my older child rather than for my own body. (C)*

Even in the busy postpartum period, they consciously attempted to improve their own dietary behavior to teach the older child good dietary habits, such as cutting back on snacks, preparing meals with healthy foods for the older child’s nutritional education, and making mealtimes fit the older child’s daily schedule.

*My three-year-old child wants a snack when I eat a snack. When I eat a meal, he eats his meal properly. He imitates me, so I decided to stop unnecessary snacks and get nutrition with my meals. (L)*

#### 3.2.2. Subjective Difficulties in Getting Information on Diet and Preparing Meals

The participants had “feeling of difficulty in getting information on diet during lactation” due to a lack of information and a mixture of different suggestions. In addition, an “increased sense of meal preparation being a burden in unique postpartum situations” was also mentioned as a barrier to healthy diet.

- “Feeling of difficulty in getting information on diet during lactation”

They had a “perception of a lack of information on the specific amount or content of a good diet during lactation”, and thus found it difficult to obtain such information and judge whether their meals were nutritionally adequate.

*I don’t think there was sufficient, detailed information about what to eat. […] I don’t think there is anything that tells me what foods to eat or what foods to avoid for breastfeeding. I wanted to get such information. (O)*

Moreover, some participants had a “feeling of difficulty in making better choices from the different suggestions about a good diet during lactation”.

*I heard that it is better to eat Washoku and avoid fatty foods. But I have also read that I don’t need to worry so much about it. There is a lot of information, so I’m not sure what is correct. (C)*

- “Increased sense of meal preparation being a burden in unique postpartum situations”

They had a “perception of a lack of time to prepare meals in the lifestyle with their babies”. They felt that it was difficult to prepare ideal meals because of being busy with housework and childcare. In reality, they ate simple meals prepared hastily while their babies were sleeping.

*I couldn’t bake or boil anything at all, so I prepared and ate something that was hassle-free. I don’t think I was very busy, but even when my baby slept I didn’t know if my baby would wake up in the next 10 min or 3 h. I cooked a few times, but I had to leave it halfway through. (E)*

*I wanted to cook more kinds of Japanese dishes with seaweed and lotus root but cooking Washoku takes a lot of time and effort, so I couldn’t prepare ideal meals. (N)*

Some participants felt a “lower priority of their own diet in their busy lives due to housework and childcare.” They did not want to spend time and effort when cooking meals just for themselves.

*I don’t want to cook (when I eat alone). I prefer to eat meals with leftovers or ready-to-eat foods. (L)*

A “perception of a lack of skills to cook Washoku, which is considered good for breast milk” made some participants find it difficult to prepare their ideal meals.

*I was advised to eat mainly Washoku (for breast milk), but I’m not so good at cooking Washoku, and I have a limited set of recipes. So I usually cooked simple dishes, such as stir fry. (B)*

They had a “sense of physical burden related to meal preparation in the course of recovery from childbirth” because they were aware of physical discomfort and fatigue.

*It was difficult to just stand up. I couldn’t stay standing as long as I thought I could, and I had pain in the pelvis and pubic bone. So I had a hard time cooking meals. (L)*

#### 3.2.3. Services and Support Regarding Their Postpartum Diet

Some participants tried to reduce the difficulty related to their diet with the “use of dietary services or convenience foods, which reduce the burden of meal preparation in postpartum life” and “dietary support from their families, especially in the postpartum period”.

- “Use of dietary services or convenience foods, which reduce the burden of meal preparation in postpartum life”

They devised ways to save time and effort in purchasing and cooking foods by “using meal kits to reduce the burden of cooking and ensure the quality of meals in a busy postpartum life,” “preparing instant food for when they could not afford to cook a meal,” and “using online grocery services to reduce the burden of going shopping.”

*(When I order from the online supermarket), I receive a meal kit and cook it at home. […] My husband isn’t so good at cooking, but he followed the recipe of the meal kit. So we could consume a lot of vegetables. I think we were able to manage our meals thanks to the meal kits. (K)*

*I was glad I had bought frozen foods. […] I always had something ready for a quick meal. (J)*

*I started using a food delivery service just after childbirth. I was trying to work out where I could make it easier for myself and save time and effort. (O)*

- “Dietary support from their families, especially in the postpartum period”

“Grocery shopping or cooking by family members” reduced the participants’ burden related to meal preparation.

*The baby is my first priority. My mother cooked meals for the first month after childbirth, so I could eat three meals regularly at the same time. (O)*

“Watching over babies by family members while cooking” also helped them concentrate on cooking.

*I could cook meals because my husband watched our baby while I cooked. (P)*

“Information on a good postpartum diet from family members” was helpful to some women when they wondered what to eat for their postpartum bodies and breastfeeding.

*My mother told me to eat Washoku and a lot of soup so I made miso soup as often as possible. (I)*

#### 3.2.4. Postpartum-Specific Appetite and Dietary Views

They recognized “changes in attitude toward diet due to pregnancy and breastfeeding”. In addition, by “viewing diet as a coping behavior for postpartum stress”, they enjoyed eating for pleasure in their busy postpartum life.

- “Changes in attitude toward diet due to pregnancy and breastfeeding”

They experienced “increased appetite due to energy expenditure by breastfeeding.” Their feeling of hunger and cravings for specific foods, such as rice, noodles, and vegetables, increased with breastfeeding, resulting in increased dietary intake.

*I’m not at all conscious of the amount of my meal. I have a great appetite and can’t stop eating. (A)*

Some participants who had stopped drinking alcohol because of breastfeeding felt “increased cravings for sweets related to giving up alcohol during lactation”.

*When I can’t drink alcohol, I want to eat sweets, and I also eat more fruits than I do when I can drink. (L)*

Their food preferences had changed during pregnancy due to pregnancy-related factors, such as nausea and vomiting, and continued unconsciously after childbirth. That was an “adoption of healthy dietary habits during pregnancy”.

*I didn’t want to eat fatty sweets, such as chocolates and whipped cream, after childbirth; I had loved them before I was pregnant. I couldn’t accept fatty sweets during pregnancy because of morning sickness. I didn’t feel like eating them even after childbirth. (K)*

However, some experienced an “increased desire for foods that were restricted during pregnancy,” including cravings for foods that they could not eat due to changes in food preferences during pregnancy, cravings for raw fish they could not eat to prevent food poisoning during pregnancy, and increased appetite due to a sense of freedom from weight gain restrictions during pregnancy.

*When I was pregnant, I had severe vomiting and ate only the same food, such as fruits and noodles. […] Just before the birth, I ate out a lot. When my mother came to help me after childbirth, I wanted her to cook something nutritious, such as Washoku or dishes with many vegetables. (G)*

*During pregnancy, I refrained from eating raw foods, such as sushi. (After childbirth), I started eating such foods more than before (perhaps as a reaction to the restraints during pregnancy). (K)*

*I didn’t want to gain weight during pregnancy, so I had tried to strongly restrict my own diet every day. It was very stressful. After childbirth, I could eat without such restrictions. Therefore, in reaction to the restrictions, I ate as much as I wanted to eat. (R)*

- “Viewing diet as a coping behavior for postpartum stress”

Participants coped with stress by “finding pleasure in meals in postpartum life with few distractions”.

*I just ate snacks, chocolates, and other ready-to-eat foods. I knew I shouldn’t eat them, but I ate them as distractions. That was because I had a sense of freedom when the baby cried and finally fell asleep. (M)*

They also mentioned “maintaining a pre-pregnancy habit of relieving stress through eating”.

*I thought I should reduce the amounts of meals and snacks I ate, but I ate them when I was stressed or tired. I wanted to reduce the amounts, but I couldn’t. […] I always relieved stress by eating. (F)*

## 4. Discussion

This study revealed the various psychosocial experiences related to dietary behavior of Japanese lactating women around one month postpartum. All participants had a [desire to have healthy meals for themselves or their families] related to their postpartum and lactation status. [Subjective difficulties in getting information on diet and preparing meals] were barriers for a healthy diet, while [services and support regarding their postpartum diet] worked as facilitators for a healthy diet by alleviating these difficulties. In addition, all participants had [postpartum-specific appetite and dietary views] due to the energy expenditure of breastfeeding, abstinence from alcohol during breastfeeding, dietary changes during pregnancy, and postpartum stress. These psychosocial experiences were influenced by physical, psychological, and social situations specific to the lactation or postpartum period, as well as by Japanese traditional customs.

They had a desire to eat healthier for the following three reasons: for their bodies, for breast milk, and for their families. For their bodies, they had a desire to have good meals for their own health and body shape. Previous studies showed that physical problems, such as mastitis, anemia, constipation, and insufficient sleep, were common in the first month postpartum [22,23,26,30,31]. The perception of these physical vulnerabilities increased our participants’ desire to prevent mastitis and their recognition of the risk of poor health, which reinforced their desire for a healthy diet. Our participants also perceived the need to be healthy to take care of their babies, which motivated them to eat healthy. In addition, some tried to adjust their diet to regain their pre-pregnancy body weight and shape. According to previous studies, 70% of postpartum Japanese women were dissatisfied with their own body shape [32], and 31% were concerned that they would not return to their pre-pregnancy weight [4]. These body image issues unique to postpartum women might lead them to have a desire to be slim, which seemed to lead to dietary behaviors, such as cutting back on sweets, reducing snacking, and avoiding late meals.

For the second reason—for breast milk—they desired a good diet for breast milk, which was similar to the findings of previous studies [1,2]. Furthermore, we found that there were two aspects of a good diet for breast milk that they considered. One was a diet that was considered good for the quality of breast milk, such as Washoku, protein-rich foods, and vegetables. The other was a diet considered necessary for adequate breast milk production, such as rice, soup, and protein-rich foods. All participants who tried to adjust their diet to produce sufficient amounts of breast milk used mixed-feeding at one month postpartum. In Japan, 88% of mixed-feeding mothers had concerns related to breastfeeding, such as a perception of low milk supply [33], and this perception may trigger changes in their lives to produce sufficient milk. A common way to increase breast milk production is through dietary content and quantity alterations; thus, lactating women often incorporate dietary adjustments into their lives [34]. Despite no clear association between these foods and the quality or quantity of breast milk, they had chosen to eat them based on their own experiences during this lactation period and information from the Internet and others around them.

Regarding the third reason—for their families—some participants expressed a desire to prepare healthier meals for their family members and eat at their families’ usual mealtimes. Most of the participants with such desires were multiparas. A previous qualitative study showed that Japanese multiparas tried to maintain the same timing of sleep, meals, and bathing for their first child after the birth of their second child [35]. Our participants also desired to maintain mealtimes that matched those of their older children. In addition, the participants had a desire to prepare good meals for their older children and husbands, even in their busy postpartum lives. A previous study [36] had also reported that the motivation for cooking meals included the desire to meet family expectations and caring for family members. This motivation was shared by our participants, indicating that they cooked healthy meals to keep their families happy and healthy even during their postpartum period. In general, motivation has been identified as an important factor in healthy dietary behavior [37]. Therefore, we believe that providing health guidance in accordance with the motivational characteristics identified in this study would help lactating women have healthy diets.

As barriers to healthy diets, our participants perceived difficulties in getting information on diet during lactation and an increased sense of meal preparation being a burden. First, the lack of specific information on what constitutes a good diet during lactation and the vast variety of suggestions made it difficult for our participants to get the information they needed. In Japan, only 20–30% of postpartum women recognized that they had received dietary guidance after childbirth [6]. Lactating women might have fewer opportunities to receive dietary guidance from healthcare providers due to the low frequency of checkups in the postpartum period and health guidance focused on childcare. As a result, lactating women seek and obtain information from the Internet, magazines, and other sources. While dietary information sources have become more diverse and much information is available, it has also become more difficult to identify correct information. Furthermore, although several studies have revealed the association between diet during the postpartum period and women’s health [25,27,38,39], there is no clear evidence of associations between diet and mastitis, milk supply, or the health of the baby. Thus, the participants may have found it difficult to obtain information on dietary habits that are considered good in the postpartum or lactation period. Even when dietary guidance from healthcare providers was available during the postpartum period, women were generally not satisfied with the guidance [40]. This may be because women assumed that there are specific foods or diets that are desirable during lactation. This assumption can lead to the discrepancy in perceptions between the women and their healthcare providers, which may contribute to women feeling that the information they were given was insufficient. Therefore, healthcare providers may need to inform lactating women that there is no clear evidence for an association between dietary intake and breast milk quantity or quality.

An increased sense of meal preparation being a burden was mentioned as the second barrier. The reasons included a lack of time, their own diet taking lower priority, physical discomfort, and not being good at cooking. Previous studies have shown that few women in the first month postpartum were able to settle into their new lifestyle patterns [41] due to reduced sleep time, physical symptoms, such as fatigue and back pain, and the strain of childcare [23,30,31,42]. Our participants also felt that they experienced physical or mental difficulties in preparing meals due to their living conditions. In addition, some participants found it difficult to prepare Washoku, which they believed to be good for their health and breast milk, due to a lack of cooking skills. In Japan, Washoku is generally perceived as a healthy diet but is difficult to prepare [43,44]. For women who consider themselves to not be good at cooking, it may be necessary to introduce services that reduce the burden of cooking or provide easy-to-prepare recipes and to tell them that they do not have to restrict themselves to Washoku or homemade dishes.

Some participants experienced a reduction in the burden of meal preparations through direct and indirect support from family members. Postpartum women with family dietary support had a significantly higher intake of some nutrients than those without any support [5]. Thus, a lack of support from family members may reduce the quality of diet by increasing the burden of meal preparation. Among our participants, family support included not only direct support in which family members were responsible for shopping and cooking, but also indirect support, such as family members watching over the baby while the participant cooked. With such indirect support, participants were able to concentrate on cooking, which resulted in reducing the burden of meal preparation. Based on the postpartum women’s needs and their family’s social background, it is necessary to adjust the type of support from family members [45,46]. Healthcare professionals may need to inform family members of postpartum women that direct as well as indirect support can be effective in alleviating the burden of postpartum women, leading to improve the quality of their diet. In addition, convenience tools, such as meal kits, instant food, and online grocery services, helped facilitate meal preparation. Meal kits are used in Japan to shorten cooking time and ensure nutritional balance [47]. In addition to the increased needs for a healthy diet due to a [desire to have healthy meals for themselves or their families], the need to reduce the burden of meal preparation due to an “increased sense of meal preparation being a burden in unique postpartum situations” may have led participants to use dietary services and convenience foods, even if they had not frequently used them in the past.

Many participants felt an increased appetite due to energy expenditure from breastfeeding. Breast milk has 60–70 kilocalories/100 mL [48], and thus lactating women must supplement the energy consumed by breastfeeding through diet. This energy expenditure associated with breast milk production would have increased the appetites of our participants. In previous studies [23,39], 86% of Japanese women felt an increase in appetite in one month postpartum and lactating women had stronger appetites than bottle-feeding women. Some participants also felt increased cravings for sweets related to giving up alcohol during lactation. A previous study of non-lactating women revealed a negative correlation between alcohol intake and the intake of sweets and sugar [49]. In addition, the craving for sweets tends to increase when people abstain from alcohol [50]. This association would also be likely in lactating women, since they are generally known to abstain from alcohol while breastfeeding.

The changes in dietary behavior during pregnancy affected postpartum diet. Approximately 70% of pregnant women experienced changes in taste and food preference [51]. Some participants experienced increased cravings for foods that they could not eat during pregnancy because of their changes in taste and food preference. In addition to such passive changes, our participants experienced active changes in dietary behavior during pregnancy. Pregnant Japanese women are aware that their own healthcare and diet affect the health and development of the fetus [52]. Over 90% of pregnant women had their own weight gain goals during pregnancy [53], and dietary restriction was often chosen as a way to appropriately control gestational weight gain [54]. Furthermore, pregnant women often refrain from eating raw fish, such as sushi and sashimi, to prevent food poisoning. Thus, the experiences of such dietary restrictions during pregnancy together with an awareness of the health of their fetus were found to influence postpartum dietary behavior. The strong desire to eat raw fish after childbirth would be due to the influence of the unique Japanese food culture that favors raw fish, such as sushi and sashimi.

Several participants viewed eating as a coping behavior for postpartum stress. Postpartum women are known to experience mental instability due to changes in lifestyle and hormonal balance. In fact, 75% of Japanese women in the first month postpartum reported feeling frustrated [23]. However, during this period, it is generally considered good to refrain from going out and reduce physical activity for recovery until one month postpartum [55]. Thus, the first month after childbirth is a time when there are few ways of coping with stress. Furthermore, women tend to view eating as a way to cope with stress [56]. For both physical and psychosocial reasons, it appears that dietary strategies to cope with stress are more likely to be chosen around the first month postpartum. In providing dietary guidance to lactating women during this period, it is important to consider not only the medical aspects but also the psychosocial aspects, such as the impact of dietary changes during pregnancy and eating as a coping strategy for stress.

The present study has three limitations. First, all participants were recruited from the respondents of a cohort study, which was conducted at a single maternity clinic located in an urban area. Thus, the results may not be relevant to situations related to the dietary behavior of women living in rural areas and of women with high-risk pregnancies. However, since the research clinic handled a large number of deliveries, it is considered that lactating women with a relatively wide range of backgrounds were included. Second, the present study might have a selection bias. However, all the women who participated in the cohort study were included in the recruitment process of the present study. Additionally, all the women who agreed to participate in the present study were interviewed. In the cohort study, the majority of women who visited the clinic and met the eligibility criteria agreed to participate. Although it is possible that women who were interested in the study were more likely to participate, other selection biases were considered to be negligible since we approached a large number of potential subjects and included all those who provided their consent. Third, the interviews were conducted at 3 months postpartum. Our participants might have had difficulty accurately recalling their experiences around the first month postpartum. However, from another perspective, we believe that the participants were able to interpret their experiences and talk about these experiences that they believe influenced their dietary behaviors.

## 5. Conclusions

The present study revealed that Japanese lactating women had various postpartum-specific psychosocial experiences related to their dietary behaviors. All participants had a [desire to have healthy meals for themselves or their families]. However, some participants had [subjective difficulties in getting information on diet and preparing meals]. As an alleviation of these difficulties, they used [services and support regarding their postpartum diet]. They also experienced [postpartum-specific appetite and dietary views] due to breastfeeding, dietary changes during pregnancy, and postpartum stress. Some of these psychosocial experiences were influenced by Japanese traditional customs in the postpartum period and familiar food preferences in the Japanese. Healthcare providers need to provide individualized dietary guidance, taking into account their physical and psychosocial circumstances, such as motivations for healthy diet, difficulties related to diet, available family support or dietary services, and appetite or view of diet.

## Figures and Tables

**Table 1:** Participants’ demographic information at one month postpartum.

Study ID	Age	Parity	Mode of Feeding	Persons Who Helped Meal Preparation	Education ^a^	Body Satisfaction
A	30–39	Multipara	Mixed ^b^	-	College	Dissatisfied
B	30–39	Primipara	Mixed ^b^	Mother	University	Neither ^d^
C	20–29	Multipara	Mixed ^b^	Husband	Graduate school	Dissatisfied
D	20–29	Primipara	Mixed ^b^	-	University	Dissatisfied
E	30–39	Primipara	Mixed ^b^	-	University	Dissatisfied
F	30–39	Multipara	Mixed ^b^	Mother	University	Dissatisfied
G	30–39	Primipara	Mixed ^b^	Mother	College	Dissatisfied
H	30–39	Multipara	Exclusive ^c^	-	College	Dissatisfied
I	20–29	Primipara	Mixed ^b^	Husband, Mother	University	Satisfied
J	30–39	Primipara	Mixed ^b^	Husband, Mother	University	Dissatisfied
K	30–39	Multipara	Mixed ^b^	Husband	University	Dissatisfied
L	30–39	Multipara	Mixed ^b^	-	College	Dissatisfied
M	30–39	Primipara	Mixed ^b^	Husband, Father-in-law	College	Dissatisfied
N	30–39	Multipara	Exclusive ^c^	Mother	University	Satisfied
O	40–49	Primipara	Mixed ^b^	Mother	University	Dissatisfied
P	30–39	Primipara	Exclusive ^c^	Husband	University	Dissatisfied
Q	40–49	Multipara	Exclusive ^c^	Mother	University	Dissatisfied
R	30–39	Multipara	Mixed ^b^	Husband	College	Dissatisfied

^a^ Most recent educational background, “College” includes College of Technology, Professional and Vocational Junior College, and Junior College. ^b^ Mixed milk feeding. ^c^ Exclusive breastfeeding. ^d^ Neither satisfied nor dissatisfied.

**Table 2:** Psychosocial experiences related to dietary behavior of lactating women.

Core Categories	Categories	Subcategories
Desire to have healthy meals for themselves or their families	Desire to have good meals for their postpartum body	Desire to prevent mastitis
		Risk perception of poor health related to childbirth or postpartum process
		Desire to remain healthy to care for their babies
		Desire to regain pre-pregnancy weight and shape
	Desire to have good meals for breast milk	Desire for the baby to drink good-quality breast milk
		Desire to produce a sufficient amount of breast milk
	Desire to prepare good meals for their family	Desire to prepare meals for the good health of their families or to maintain regular mealtimes for their families
Subjective difficulties in getting information on diet and preparing meals	Feeling of difficulty in getting information on diet during lactation	Perception of a lack of information on the specific amount or content of a good diet during lactation
		Feeling of difficulty in making better choices from the different suggestions about a good diet during lactation
	Increased sense of meal preparation being a burden in unique postpartum situations	Perception of a lack of time to prepare meals in the lifestyle with their babies
		Lower priority of their own diet in their busy lives due to housework and childcare
		Perception of a lack of skills to cook Washoku *, which is considered good for breast milk
		Sense of physical burden related to meal preparation in the course of recovery from childbirth
Services and support regarding their postpartum diet	Use of dietary services or convenience foods, which reduce the burden of meal preparation in postpartum life	Using meal kits to reduce the burden of cooking and ensure the quality of meals in a busy postpartum life
		Preparing instant food for when they could not afford to cook a meal
		Using online grocery services to reduce the burden of going shopping
	Dietary support from their families, especially in the postpartum period	Grocery shopping or cooking by family members
		Watching over babies by family members while cooking
		Information on a good postpartum diet from family members
Postpartum-specific appetite and dietary views	Changes in attitude toward diet due to pregnancy and breastfeeding	Increased appetite due to energy expenditure by breastfeeding
		Increased cravings for sweets related to giving up alcohol during lactation
		Adoption of healthy dietary habits during pregnancy
		Increased desire for foods that were restricted during pregnancy
	Viewing diet as a coping behavior for postpartum stress	Finding pleasure in meals in postpartum life with few distractions
		Maintaining a pre-pregnancy habit of relieving stress through eating

* Washoku: Japanese traditional cuisine.

## Data Availability

Not applicable.

## References

[1] Tsukada Y., Kawashima A., Ota M., Yoshimura K., Kuchiishi R., Kamezaki A., Tanaka M. (2017). Infant-feeding difficulties and coping techniques in mothers within one month after hospital discharge. Jpn. J. Matern. Health.

[2] Sakamoto M., Matsuoka M., Misumi J., Shimizu K. (2002). Dietary habits and influencing factors among postpartum mothers in the first month postpartum. Jpn. J. Matern. Health.

[3] Inome A., Inoue N., Yoshidome A. (2020). The self-care activities of mothers who lives in remote locations without childbearing facilities during gestation and childbirth. J. Jpn. Acad. Midwifery.

[4] Tsutsumi C., Takano A., Mitsuhashi F. (2007). Study on Enhanced Support for Women’s Dietary Habits during the Pregnancy and Lactation Periods (I)—The current state of women’s dietary habits before and after giving birth. Rep. Stud. Jpn. Child Fam. Res. Inst..

[5] Matsueda M., Takahashi K., Sato Y., Kaneshige E. (2000). A study of nutritional guidance during pregnancy and postpartum period. Jpn. J. Matern. Health.

[6] Kamezaki A., Tanaka M., Nakamura E., Hara R. (2015). The actual situation of the eating habits and the nutrition situation of mothers with infants. Jpn. J. Matern. Health.

[7] Shiraishi M., Matsuzaki M., Tsunematsu R., Watanabe S., Kobayashi R., Haruna M. (2021). Effects of Individual Dietary Intervention on Nutrient Intake in Postpartum Japanese Women: A Randomized Controlled Trial. Nutrients.

[8] MacMillan Uribe A.L., Olson B.H. (2019). Exploring Healthy Eating and Exercise Behaviors Among Low-Income Breastfeeding Mothers. J. Hum. Lact..

[9] Chang M.W., Nitzke S., Guilford E., Adair C.H., Hazard D.L. (2008). Motivators and barriers to healthful eating and physical activity among low-income overweight and obese mothers. J. Am. Diet. Assoc..

[10] Snyder K., Pelster A.K., Dinkel D. (2020). Healthy eating and physical activity among breastfeeding women: The role of misinformation. BMC Pregnancy Childbirth.

[11] Christenson A., Johansson E., Reynisdottir S., Torgerson J., Hemmingsson E. (2016). Women’s Perceived Reasons for Their Excessive Postpartum Weight Retention: A Qualitative Interview Study. PLoS ONE.

[12] Faria-Schützer D.B., Surita F.G., Rodrigues L., Turato E.R. (2018). Eating Behaviors in Postpartum: A Qualitative Study of Women with Obesity. Nutrients.

[13] Teh K., Quek I.P., Tang W.E. (2021). Postpartum dietary and physical activity-related beliefs and behaviors among women with recent gestational diabetes mellitus: A qualitative study from Singapore. BMC Pregnancy Childbirth.

[14] Versele V., Stok F.M., Aerenhouts D., Deforche B., Bogaerts A., Devlieger R., Clarys P., Deliens T. (2021). Determinants of changes in women’s and men’s eating behavior across the transition to parenthood: A focus group study. Int. J. Behav. Nutr. Phys. Act..

[15] Sundarapperuma T.D., Wijesinghe C.J., Hettiarachchi P., Wasalathanthri S. (2018). Perceptions on Diet and Dietary Modifications during Postpartum Period Aiming at Attenuating Progression of GDM to DM: A Qualitative Study of Mothers and Health Care Workers. J. Diabetes Res..

[16] Murray-Davis B., Grenier L., Atkinson S.A., Mottola M.F., Wahoush O., Thabane L., Xie F., Vickers-Manzin J., Moore C., Hutton E.K. (2019). Experiences regarding nutrition and exercise among women during early postpartum: A qualitative grounded theory study. BMC Pregnancy Childbirth.

[17] Cantarero L., Espeitx E., Gil Lacruz M., Martín P. (2013). Human food preferences and cultural identity: The case of Aragón (Spain). Int. J. Psychol..

[18] Sato W., Rymarczyk K., Minemoto K., Wojciechowski J., Hyniewska S. (2019). Cultural Moderation of Unconscious Hedonic Responses to Food. Nutrients.

[19] Raman S., Nicholls R., Ritchie J., Razee H., Shafiee S. (2016). Eating soup with nails of pig: Thematic synthesis of the qualitative literature on cultural practices and beliefs influencing perinatal nutrition in low and middle income countries. BMC Pregnancy Childbirth.

[20] Nagatsuru M. (2006). Influence of mother-daughter relationship during the perimtal period on the mental health of puerperas. Jpn. J. Matern. Health.

[21] Minami T., Ohara T., Mutou Y. (2006). Childbirth and child care support for mother -focus on the experience of “ChildbirthSatogaeri”. J. Home Econ. Jpn..

[22] Riordan J.M., Nichols F.H. (1990). A descriptive study of lactation mastitis in long-term breastfeeding women. J. Hum. Lact..

[23] Shinkawa H. (2021). Changes in pregnancy-related discomforts in women from the third-trimester pregnancy to 1 year postpartum. J. Jpn. Acad. Midwifery.

[24] Keikha M., Bahreynian M., Saleki M., Kelishadi R. (2017). Macro- and Micronutrients of Human Milk Composition: Are They Related to Maternal Diet? A Comprehensive Systematic Review. Breastfeed. Med..

[25] Hoshino A., Yamada A., Tanabe R., Noda S., Nakaoka K., Oku Y., Katayama C., Haraikawa M., Nakano H., Harada M. (2017). Relationships between bone mass and dietary/lifestyle habits in Japanese women at 3–4 months postpartum. Public Health.

[26] Kobiyama A., Suzuki E., Takayama Y. (2015). Post-partum anemia and factors that work against alleviation of the anemia. Jpn. J. Nurs. Sci..

[27] Tsubouchi M., Tokudome Y., Machida K., Shida N., Iwata K., Mizukami K. (2019). Relationship between the Consumption of n-3 Polyunsaturated Fatty Acids and Mental Health during Late Pregnancy and Postpartum. J. Jpn. Soc. Shokuiku.

[28] Sandelowski M. (2000). Whatever happened to qualitative description?. Res. Nurs. Health.

[29] Lincoln Y.S., Guba E.G. (1985). Naturalistic Inquiry.

[30] Hayase M., Shimada M., Inui T., Samejima M., Tamotsu S., Shinkawa H., Ogata T., Tokimoto A., Hojoh M. (2008). Relationships between bone mass and dietary/lifestyle habits in Japanese women at 3–4 months postpartum. Perinat. Med..

[31] Inui T., Shimada M., Hayase M., Ogata T., Tokimoto A., Hojoh M., Shinkawa H. (2008). Longitudinal study on changes of sleep-wake rhythm in mothers from late pregnancy to 4 months postpartum using sleep log. J. Jpn. Acad. Midwifery.

[32] Maruyama K., Ohyama K., Suzuki K., Yamagata Z. (2015). Influences on degree of satisfaction with body type during pregnancy. Jpn. J. Matern. Health.

[33] Ministry of Health, Labor and Welfare Summary Report of National Nutrition Survey on Preschool Children. https://www.mhlw.go.jp/file/06-Seisakujouhou-11900000-Koyoukintoujidoukateikyoku/0000134207.pdf.

[34] Akiyama-Sannomaru Y., Maki M., Takeuchi H., Himi T., Fukaya M., Saimei M., Kobayashi M. (2012). Effects of Nursing and Eating Habits of Mothers in the Lactation Period. J. Jpn. Soc. Breastfeed. Res..

[35] Yamazaki A. (2003). The formation of a family of four while considering the development of the firstborn child. J. Jpn. Acad. J. Midwifery.

[36] Kamata H., Ando S. (2014). Development of a meal-making motivation scale for homemakers. Jpn. J. Health Educ. Promot..

[37] Michaelidou N., Christodoulides G., Torova K. (2012). Determinants of healthy eating: A cross-national study on motives and barriers. Int. J. Cosumer Stud..

[38] Yamamoto N., Nishimura T., Akahoshi E. (2014). Factors associated with infant feeding practices in mothers at four months after delivery. Nagasaki Med. J..

[39] Kawakami T., Murota I., Nakama M. (2006). The quality of health guidance during labor stage and postpartum period: From a survey on the satisfaction of mothers. Teishin Igaku.

[40] Jiang W., Mo M., Li M., Wang S., Muyiduli X., Shao B., Jiang S., Yu Y. (2018). The relationship of dietary diversity score with depression and anxiety among prenatal and post-partum women. J. Obs. Gynaecol. Res..

[41] Benesse Research Institute Survey of Prenatal and Postpartum Life and Support. https://berd.benesse.jp/up_images/research/kisoshukei.pdf.

[42] Nohara M., Nakata H. (2019). A Follow-up Study on Worries in Childcare and Quality of Life Experienced by Mothers with First Children. Pediatr. Health Res..

[43] Hirai K., Asano M., Kawakami E., Hosotani K., Ohno Y., Higuchi H., Maeda A. (2001). Awareness of Health and Life Style among Adolescents. J. Integr. Study Diet. Habits.

[44] Takahashi S. (2002). Attitude toward diet among junior college students: Focusing on wasyoku. Bull. Morioka Daigaku Jr. Coll..

[45] Sawada H., Sekizuka-Kagami N., Ota Y., Maida Y. (2020). Mothers’ child-rearing stress and coping behaviors from one month postpartum to four months after childbirth. J. Jpn. Acad. Nurs. Sci..

[46] Kubota T., Kuroiwa A. (2021). Care needs for midwives that primiparas one month after delivery feel about childcare. J. Jpn. Matern. Infant Care Assoc..

[47] MMD Research Institute Survey on Meal Kit Usage. https://mmdlabo.jp/investigation/detail_1969.html.

[48] Isomura H., Ochiai F., Saimoto M., Shimizu Y., Kitamura K. (2007). Studies on Japanese breast milk composition. Jpn. J. Matern. Health.

[49] Colditz G.A., Giovannucci E., Rimm E.B., Stampfer M.J., Rosner B., Speizer F.E., Gordis E., Willett W.C. (1991). Alcohol intake in relation to diet and obesity in women and men. Am. J. Clin. Nutr..

[50] Alarcon R., Tiberghien M., Trouillet R., Pelletier S., Luquiens A., Ahmed S.H., Nalpas B., Alaux-Cantin S., Naassila M., Perney P. (2021). Sugar intake and craving during alcohol withdrawal in alcohol use disorder inpatients. Addict. Biol..

[51] Mito H., Ishida M., Sakai M., Takasugi H., Nishiguchi A., Yoshikawa S., Ozasa S. (2019). Changes in taste, food preferences, and sense of smell during pregnancy. Osaka J. Matern. Health.

[52] Manabe E., Seto M., Agari I., Kitagawa M., Kotake K. (2001). Psychological Factors related to Motivating Self-Care Behaviors in First-Time Pregnant Women. Jpn. J. Matern. Health.

[53] Takimoto H., Mitsuishi C., Kato N. (2011). Attitudes toward pregnancy related changes and self-judged dieting behavior. Asia Pac. J. Clin. Nutr..

[54] Fujiya R., Ishizuka N. (2008). Evaluation of Weight Management Guidance in Mothers’ Classes: From Gestational Weight Gain and Pregnant Women’s Desires. J. Jpn. Nurs. Assoc.-Matern. Nurs..

[55] Nagatsuru M., Miyasato K. (2001). A research of folk care regarding the movement of the postpartum women: From advice given by non-professionals. Jpn. J. Matern. Health.

[56] Fukagawa T., Hashimoto M., Nonomura M. (2022). First survey on the relationship between stress and dietary habit during the COVID-19-related state of emergency. J. Jpn. Mibyou Assoc..

